# Smooth and Rough Biotypes of *Arcanobacterium haemolyticum* Can Be Genetically Distinguished at the Arcanolysin Locus

**DOI:** 10.1371/journal.pone.0137346

**Published:** 2015-09-18

**Authors:** Haley S. Ruther, Kalyn Phillips, Dolores Ross, Alyssa Crawford, M. Payton Weidner, Osama Sammra, Christoph Lämmler, David J. McGee

**Affiliations:** 1 Department of Microbiology & Immunology, Louisiana State University Health Sciences Center, Shreveport, Louisiana, United States of America; 2 Southwood High School, Shreveport, Louisiana, United States of America; 3 Department of Chemistry, Centenary College of Louisiana, Shreveport, Louisiana, United States of America; 4 Institut für Pharmakologie und Toxikologie,Justus-Liebig-Universität, Gießen, Germany; Belgian Nuclear Research Centre SCK•CEN, BELGIUM

## Abstract

*Arcanobacterium haemolyticum* is a Gram-positive, β-hemolytic emerging human pathogen that is classified into smooth or rough biotypes. This bacterial species is also a rare pathogen of animals. Smooth biotypes possess smooth colony edges, are moderate to strong in β-hemolysis, and predominately cause wound infections. In contrast, rough biotypes possess rough and irregular colony edges, have weak to no β-hemolytic activity, and predominately cause pharyngitis. Using horse erythrocytes we confirmed that smooth isolates are generally more hemolytic than rough isolates. A hemolysin from *A*. *haemolyticum*, arcanolysin (*aln*/ALN), was recently discovered and is a member of the cholesterol-dependent cytolysin (CDC) family. PCR amplification of *aln* from all 36 smooth *A*. *haemolyticum* isolates yielded the expected 2.0 kb product. While 21 rough isolates yielded the 2.0 kb product, 16 isolates had a 3.2 kb product. The extra 1.2 kb segment was 99% identical to IS*911* (insertion sequence) from *Corynebacterium diphtheriae*. PCR amplification and sequence analysis of the upstream region of *aln* revealed ~40 nucleotide polymorphisms among 73 clinical isolates from Finland, Denmark, Germany and United States (Nebraska). Remarkably, multi-sequence alignments of the *aln* upstream region demonstrated that ~90% of the isolates phylogenetically clustered as either smooths or roughs. Differential restriction enzyme analysis of the *aln* upstream region also demonstrated that the *aln* upstream region of most (~75%) smooth isolates was cleaved with *Cla*I while this region in most (~86%) rough isolates was cleaved with *Xcm*I. We conclude that the *aln* upstream region can be used to genetically distinguish between smooth and rough biotypes of this important emerging pathogen.

## Introduction

*Arcanobacterium haemolyticum* is a Gram-positive, β-hemolytic, pleomorphic rod that can cause a wide range of diseases in humans, from pharyngitis and wound infections in immunocompetent patients to more severe invasive diseases in immunocompromised patients [1–5]. It can also be rarely isolated as a pathogen in animals [6, 7] This emerging pathogen is classified into two biotypes based on biochemical, colony morphology, hemolytic activity and disease association [8, 9]. Rough isolates are β-glucuronidase positive, possess a rough and irregular edge, have weak to no β-hemolysis, and are associated with pharyngitis. In contrast, smooth isolates are β-glucuronidase negative, possess smooth edges, are moderate to strong in β-hemolysis, and are associated with wound infections [9]. These two main biotypes of *A*. *haemolyticum* cannot currently be distinguished genetically and why rough isolates have weaker β-hemolysis is unknown. Weaker hemolysis by some clinical isolates, especially on sheep blood, may partially explain why the organism is missed in clinical specimens.

*A*. *haemolyticum* has been known to have hemolytic activity since 1946 [1], yet no bona fide hemolysin had been reported until recently. In 2011, arcanolysin (*aln/*ALN), a member of the cholesterol-dependent cytolysin (CDC) toxin family was identified [10]. More than 20 Gram-positive bacterial species produce CDCs, which are a family of pore-forming toxins. Some well-characterized members include: listeriolysin O (LLO), perfringolysin O (PFO), streptolysin O (SLO), and pneumolysin (PLY) [11]. CDCs are produced and secreted as monomers. Upon contact with the eukaryotic membrane, CDCs oligomerize and form a large β-barrel pore, allowing for loss of amino acids, nucleotides, ions and large molecules. Pore formation results in lysis of the cell.

We determined the genetic variability within *aln* in *A*. *haemolyticum* clinical isolates from Finland, Denmark, Germany and United States (Nebraska). PCR amplification and sequence analyses of *aln* from 73 smooth and rough *A*. *haemolyticum* clinical isolates were conducted. We demonstrate that some rough isolates possess a 1.2 kb insertion sequence element within the *aln* coding region. We also provide evidence that smooth and rough biotypes can be genetically distinguished by both the sequence of the upstream region of *aln* and by differential restriction enzyme cleavage patterns. Molecular distinction between smooth and rough biotypes of *A*. *haemolyticum* may lead to improved diagnosis of this important emerging bacterial pathogen.

## Materials and Methods

### Bacterial strains and growth conditions

All 73 bacterial strains used in this study are listed in Table 1. *A*. *haemolyticum* strain ATCC 9345 is the type strain for this organism [1]. All other *A*. *haemolyticum* strains used in this study are clinical isolates from sinusitis, pharyngitis, wound infections, abscesses, and bacteremia cases. *A*. *haemolyticum* was grown on Todd-Hewitt (TH) agar plates supplemented with 6% defibrinated horse blood (Quad 5, Ryegate, MT) in 5% CO_2_ for 48 hours.

**Table 1:** *Arcanobacterium haemolyticum* strains used in this study.

Strain	Biotype	IS Element	Restriction Pattern	Disease	Source^2^
	(S or R) ^1^	(Y or N)	(C, X, N)		
AhS1	S	N	C	Wound infection	PC
AhS2	S	N	C	Paronychia	PC
AhS3	S	N	C	Wound infection	PC
AhS4	S	N	C	Infected leg ulcer	PC
AhS5	S	N	C	Wound infection	PC
AhS6	S	N	C	Wound infection	PC
AhS7	S	N	C	Infected leg ulcer	PC
AhS8	S	N	C	Wound infection	PC
AhS9	S	N	C	Wound infection	PC
AhS10	S	N	C	Paronychia	PC
AhS11	S	N	C	Pharyngitis	PC
AhS12	S	N	C	Pharyngitis	PC
AhS13	S	N	X	Pharyngitis	PC
AhS14	S	N	C	Pharyngitis	PC
AhS15	S	N	X	Pharyngitis	PC
AhS16	S	N	C	Sinusitis	PC
AhS17	S	N	C	Sinusitits	PC
AhS18	S	N	C	Pharyngitis	PC
AhS19	S	N	X	Pharyngitis	PC
AhS20	S	N	C	Pharyngitis	PC
AhR21	R	N	X	Peritonsillar abscess	PC
AhS22	S	N	C	Pharyngitis, pneumonia	PC
AhS23	S	N	C	Diabetic foot gangrene	PC
AhS24	S	N	N	Tonsillitis	PC
AhS25	S	N	C	Metatarsal osteitis	PC
AhR26	R	Y	X	Wound infection	PC
AhR27	R	Y	X	Wound infection	PC
AhR28	R	Y	X	Pharyngitis	PC
AhR29	R	N	C	Peritonsillar abscess	PC
AhR30	R	N	X	Sinusitis	PC
AhR31	R	Y	X	Peritonsillar abscess	PC
AhR32	R	N	X	Peritonsillar abscess	PC
AhR33	R	Y	X	Pharyngitis	PC
AhR34	R	Y	X	Pharyngitis	PC
AhR35	R	N	X	Pharyngitis	PC
AhR36	R	Y	X	Pharyngitis	PC
AhR37	R	Y	X	Peritonsillar abscess	PC
AhR38	R	Y	X	Wound infection	PC
AhR39	R	Y	X	Pharyngitis	PC
AhR40	R	Y	X	Pharyngitis	PC
AhR41	R	Y	X	Pharyngitis	PC
AhR42	R	Y	X	Pharyngitis	PC
AhR43	R	Y	X	Pharyngitis	PC
AhR44	R	N	X	Pharyngitis	PC
AhR45	R	N	X	Pharyngitis	PC
AhR46	R	Y	X	Pharyngitis	PC
AhR47	R	N	X	Pharyngitis	PC
AhR48	R	N	N	Pharyngitis	PC
AhR49	R	Y	X	Pharyngitis	PC
AhR50	R	N	X	Pharyngitis	PC
B0961-98	R	N	X	Pharyngitis	PI
B1025-98	S	N	X	Pharyngitis	PI
B1088-99	R	N	X	Pharyngitis	PI
B2953-00	R	N	X	Pharyngitis	PI
B4635-01	R	N	X	Pharyngitis	PI
B4636-01	S	N	X	Pharyngitis	PI
B5229-01	S	N	N	Pharyngitis	PI
B5366-01	S	N	C	Pharyngitis	PI
B5495-02	R	N	X	Pharyngitis	PI
B5646-02	R	N	X	Pharyngitis	PI
B5813-02	S	N	N	Blood	PI
B2130-12	R	N	X	Pharyngitis	PI
B2492-12	R	N	X	Pharyngitis	PI
ATCC9345	S	N	C		ATCC reference strain
7–2596628	R	N	C	Periappendicular abscess	CL
7–4438845	R	N	C	Osteomyelitis	CL
7–4823400	S	N	C	Abscess	CL
7–4991567	S	N	C	Abscess	CL
E2-1797395	S	N	C	Heel	CL
E2-1942131	S	N	C	Osteomyelitis	CL
P646^3^	R	N	C	Nasal discharge	CL
P5648/10^4^	S	N	N	Wound infection	CL
2289/09^4^	S	N	C	Castrational wound	CL

^1^ Abbreviations: ATCC = American Type Culture Collection. S = smooth biotype (n = 36), R = rough biotype (n = 37). IS element refers to presence (Y, yes) or absence (N, no) of IS within *aln* coding region. C, *Cla*I site, X, *Xcm*I site, N, neither *Cla*I nor *Xcm*I sites.

^2^ PC, Petteri Carlson isolates, Finland; PI, Peter C. Iwen isolates, United States (Nebraska); CL, Christoph Lämmler isolates, Germany and Denmark.

^3^ Donkey isolate.

^4^ Horse isolate.

### DNA techniques

All oligonucleotide primers used in this study are listed in Table 2 and were purchased from Integrated DNA Technology (IDT, Coralville, IA). PCR amplification was performed using GoTaq (Promega, Madison, WI) with supplied reaction buffer for 30 cycles consisting of 5 minutes at 94°C, 1 minute at 55°C, and either 1 minute (upstream region of *aln*, amplicon size: 830 bp) or 2 minutes (*aln*-coding region, amplicon size: 2.0 kb) at 72°C, with a final extension at 72°C for 5 minutes. Restriction enzyme analysis of the upstream region of *aln* amplicon was performed using *Cla*I or *Xcm*I (New England Biolabs, Ipswich, MA) at 37°C for 60 minutes. DNA agarose gel electrophoresis was performed as described [12].

**Table 2:** Oligonucleotide primers used in this study.

Gene	Primer Name	Sequence (5`-3`)	Amplicon (bp)
*aln*	DM1014 (Forward)	5`-TCCCCGCGGTCAAGTTATGCCGGGAATG-`3	1,992^1^
*aln*	DM1015 (Reverse)	5`-CCGATCGATGTTCTTGAACCAAGG-`3	
Upstream region of *aln*	DM1078 (Forward)	5`-ACATGCTTCAAGGGATGGAA-`3	830
Upstream region of *aln*	DM1080 (Reverse)	5`-TTTCAGTTGCGGAAAGGTT-`3	

^1^ Product size is 1.2 kb larger in some rough isolates.

### Hemolysis Assay

Horse erythrocytes were gently washed at least three times in 1X phosphate-buffer saline (PBS), centrifuged at 2,000 rpm (500 x g) for 10 minutes at room temperature and resuspended in 1X PBS. In a 96-well plate, horse erythrocytes were mixed with resuspended bacteria in a 1:1 ratio and incubated at 37°C for 60 minutes. The plate was centrifuged at 4,000 rpm (1,500 x g) for 10 minutes at 25°C and the supernatants were removed and absorbance was measured at 415 nm. Positive and negative controls were 1% Triton-X-100 and 1X PBS, respectively. Percent hemolysis (% hemolysis) was calculated using the following equation: (Sample-1X PBS)/(Triton-X-100 – 1X PBS) x 100.

### Computer analysis

Sequencing reactions were performed by the DNA sequencing facility at Arizona State University. All sequence data were compiled on Microsoft Word and database searches were performed using Blastn. Multi-sequence alignments were performed using ClustalO 2.1 (www.ebi.ac.uk/tools/msa/ClustalO/.) Trees were drawn using TreeView [13].

### GenBank

Nucleotide sequences were deposited in GenBank under accession numbers KP668885—KP668957.

### Statistics

Comparison of nucleotides in the upstream region of smooth versus rough biotypes were conducted in a contingency table using two-sided Fisher’s exact test. Hemolysis assays were compared using a t test. *P* < 0.05 was considered significant.

## Results

### Colony morphology and hemolytic activity of clinical isolates of *A*. *haemolyticum* on Todd-Hewitt media supplemented with horse blood

**Fig 1:**
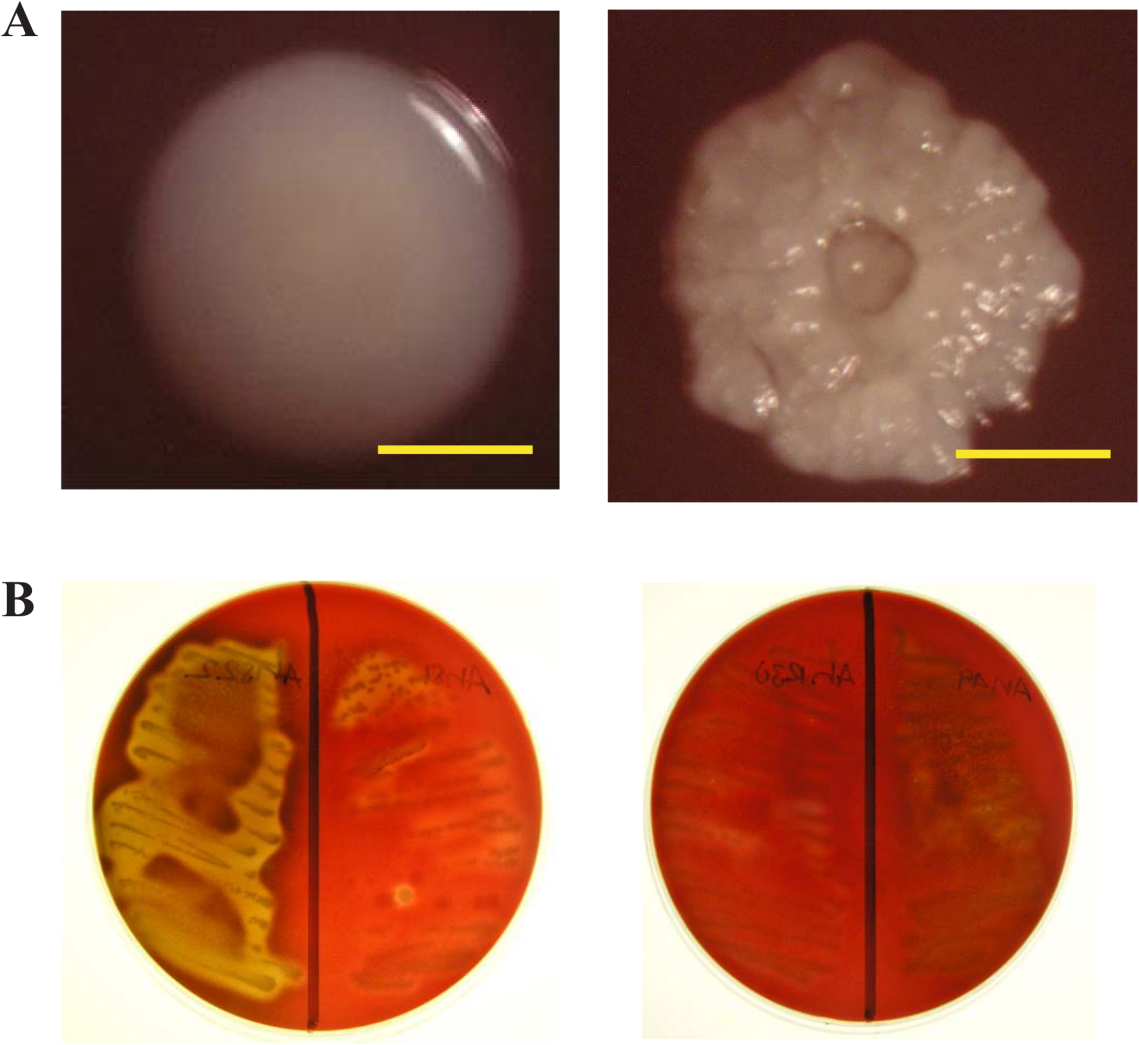
Colony morphology and hemolysis of clinical isolates of *A*. *haemolyticum* is enhanced on Todd-Hewitt media supplemented with 6% horse blood. Growth of clinical isolates for 48 hours in 5% CO_2_ showcases the differences between colony morphology and hemolysis of *A*. *haemolyticum*. (A) Distinct colony morphologies between smooth (Left-S1) and rough (Right-R44) isolates visualized after 48 h incubation. Line represents 1.0 mm. (B) Hemolysis variation between smooth (Left-S1 and S22) and rough (Right-R49 and R30) isolates.

In 1994, Carlson *et al*. recognized two biotypes of *A*. *haemolyticum* [9]. Based on biochemical, colony morphology, and hemolytic activity, the two biotypes were designated as either rough or smooth [9]. A direct visualization of *A*. *haemolyticum* is not well published, and attempts to visualize morphologies of clinical isolates of *A*. *haemolyticum* as described by Carlson were unsuccessful. However, utilization of Todd-Hewitt (TH) media supplemented with 6% horse blood allowed for visualization of the smooth and rough morphology of *A*. *haemolyticum* in our laboratory (Fig 1A). Likewise, the hemolytic phenotype of various isolates was observed on this media, and was enhanced from smooth isolates in comparison with rough isolates (Fig 1B).

### PCR amplification and sequence analysis of the *aln*-coding region uncovers an insertion sequence element within the *aln* open-reading frame

**Fig 2:**
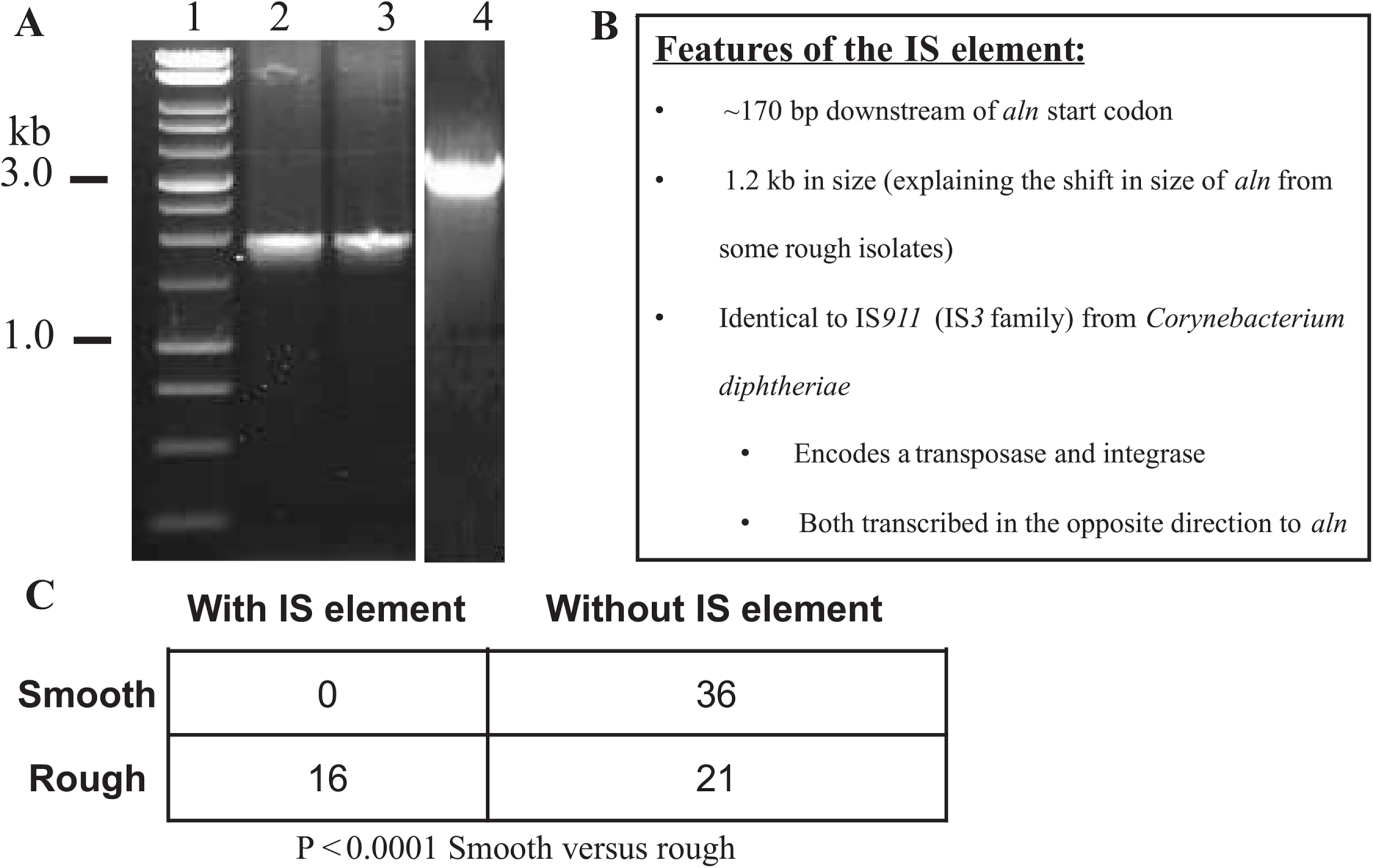
PCR and sequence analysis of the *aln*-coding region yields an insertion sequence (IS) element in some rough isolates of *A*. *haemolyticum*. The *aln*-coding region of rough and smooth clinical isolates of *A*. *haemolyticum* was PCR amplified using primers DM1014 and DM1015. (A) DNA gel electrophoresis of smooth isolate ATCC9345 (Lane 2) and rough isolate AhR30 (Lane 3) amplify the predicted 2.0 kb product compared to rough isolate, AhR28 (Lane 4), which amplifies a 3.2 kb product. (B) Features of the IS element found in the *aln* coding region of some rough isolates of *A*. *haemolyticum*. (C) Statistical analysis of *A*. *haemolyticum* isolates from Finland or United States (Nebraska).

*A*. *haemolyticum* exists in two distinct biotypes. Smooth isolates possess moderate to strong β-hemolysis, while rough isolates have weak to no β-hemolytic activity. We hypothesized that this variability in hemolytic activity could be due to differences in the *aln*-coding region from clinical isolates of *A*. *haemolyticum*. To test this, PCR amplification of the *aln* open-reading frame (ORF) from smooth isolate ATCC 9345 and rough isolates (AhR30 and AhR28) were compared on a 0.8% agarose gel. Both ATCC 9345 and rough isolate AhR30 PCR products were at the expected 2.0 kb size. Surprisingly, we obtained a 3.2 kb PCR product from rough isolate AhR28 (Fig 2A). Sequence analysis of this 3.2 kb amplicon revealed an insertion sequence (IS) element that has 99% nucleotide sequence identity to a transposase and integrase from IS*911 Corynebacterium diphtheriae* (GenBank Accession #CP003215.1).[14]. *IS911* is a member of the IS*3* family of transposable elements [15]. In the *A*. *haemolyticum* genome there are seven complete IS*911* copies (1.25 kb) all 99–100% identical to each other and four partial copies (322–323 bp, 71% identical to the full copies). The insertion within the *aln* coding region in some rough isolates was a complete IS*911* copy. The two IS open-reading frames are in the opposite direction to the *aln* ORF and is inserted ~170 bp downstream of the *aln* start codon (Fig 2B). When all Finland clinical isolates were screened for presence of the IS element within *aln*, we found that none of the smooth isolates possessed this IS element, while half of the rough isolates did (Fig 2C). The insertion of the IS element was determined to be in the same *aln* location in rough isolates. Interestingly, none of the United States (Nebraska), German or Denmark isolates contained this IS element within the *aln* coding region, suggesting that the geographical location may impact the acquisition of this IS element.

### Hemolytic activity of smooth and rough isolates of *A*. *haemolyticum* incubated with horse erythrocytes

**Fig 3:**
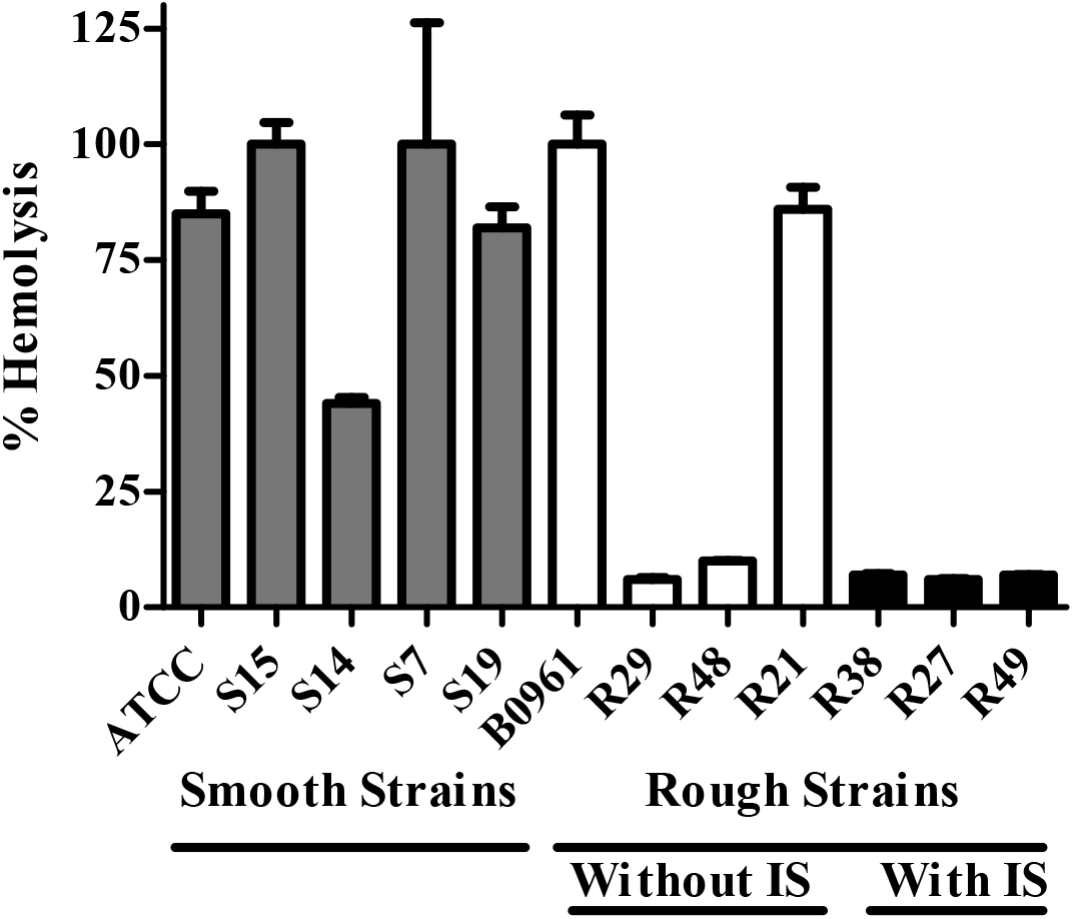
Hemolytic activity of smooth and rough isolates of *A*. *haemolyticum* incubated with horse erythrocytes. Hemolysis assay on washed horse erythrocytes (1%) incubated with smooth or rough live strains of *A*. *haemolyticum*. *A*. *haemolyticum* smooth and rough isolates vary in hemolytic activity when bacteria are incubated with horse erythrocytes for one hour. Representative of at least six individual experiments. Shown is an average of three replicates ± standard deviation. Smooth isolates tend to have more hemolytic activity than rough isolates.

Since the IS element is located ~170 bp downstream of the *aln* start codon, which would presumably disrupt the *aln* coding region, this finding may explain why some rough isolates have low hemolytic activity. Incubation of five smooth and seven rough isolates of *A*. *haemolyticum* with horse red blood cells resulted in various hemolytic activities (Fig 3). While 4/5 of smooth isolates had near maximal hemolysis, AhS14 had lower hemolytic activity. Interestingly 5/7 of the rough isolates had hemolytic activity less than 10% of the positive control. All isolates tested that had the IS element inserted into the *aln* coding region (R27, R38, R49) were very low in hemolytic activity. While it is currently unknown whether the IS element directly affects hemolytic activity, there are rough isolates that lack the IS element within the *aln* coding region (R29, R48) that also have low hemolytic activity (Fig 3). Thus, there may be additional variables dampening hemolytic activity in rough isolates. Overall, we conclude that hemolytic activity varies in clinical isolates and this corresponds to earlier observations that smooth isolates tend to have strong hemolysis, whereas rough isolates tend to have weak to no hemolysis [9].

### The upstream region of *aln* yields nucleotide polymorphisms that allow molecular distinction between rough and smooth biotypes of *A*. *haemolyticum*

Other than the IS element within *aln* in some rough isolates, we did not observe any major sequence variations within the *aln* coding region of several strains examined However, we did not sequence the *aln* coding region in all isolates. We therefore focused on the upstream region of *aln* to see if it varied and correlated with the colony morphology of *A*. *haemolyticum*. PCR amplification of the intergenic region resulted in a 830 bp product, which amplified in all clinical isolates of *A*. *haemolyticum* from Finland, Denmark, Germany and United States (Nebraska). This PCR product encompasses the 3`end of phosphoglycerate mutase, a tRNA-ala gene, the Shine-Dalgarno (SD) sequence, and the 5`end of *aln*. Sequence analysis of the upstream region revealed several characteristics that have not been reported.

**Fig 4:**
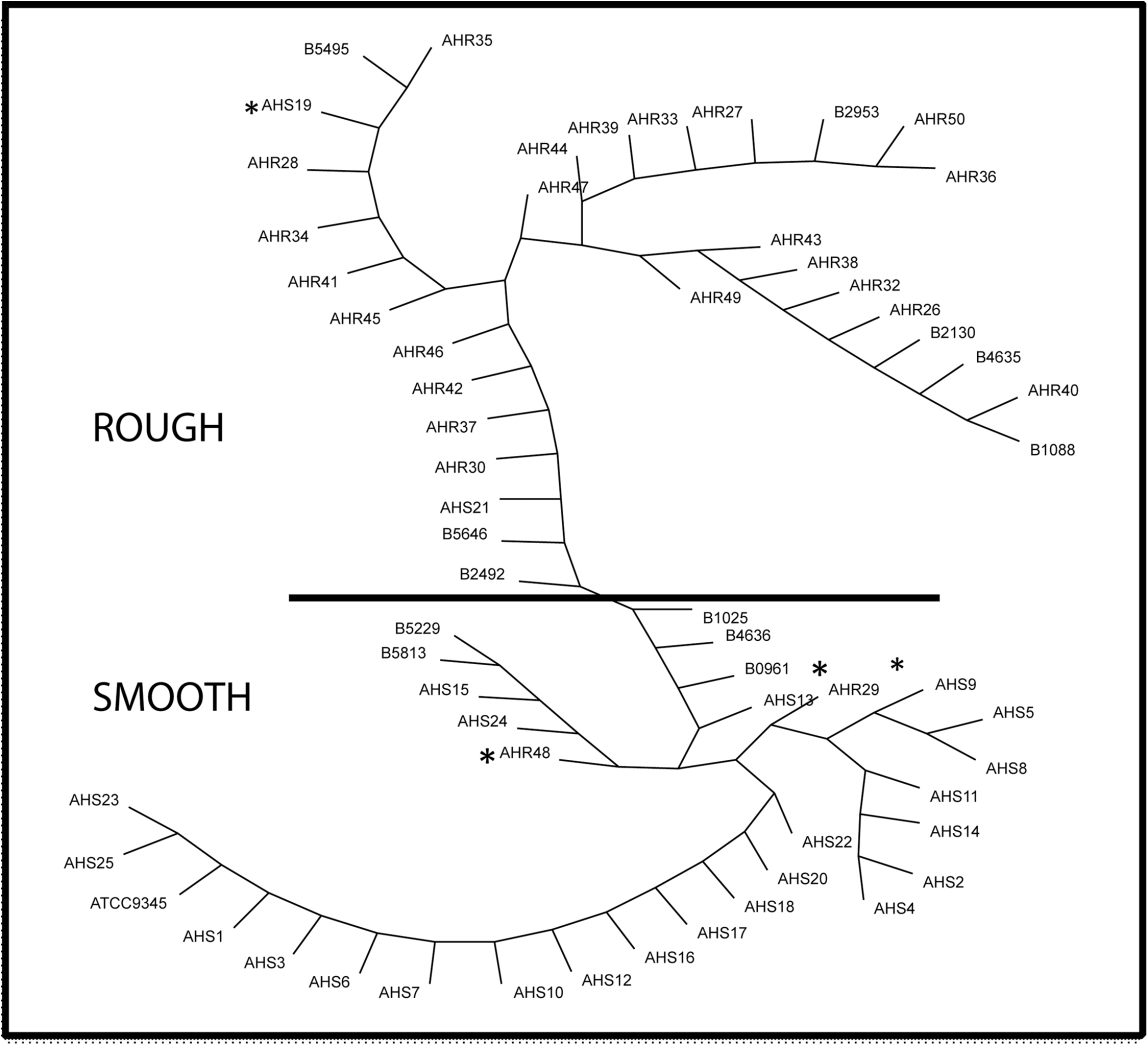
Smooth and rough clinical isolates of *A*. *haemolyticum* can be distinguished by sequence analysis of the upstream region of *aln*. The upstream region of *aln* from 73 clinical isolates was PCR-amplified using primers DM1078 and DM1080 to generate an ~830 bp product. Following sequence analysis, ~ 50 bp of the 5’ end and ~70 bp of the 3’ end were trimmed, yielding a ~715 bp upstream region for further alignments and analysis. An unrooted tree (generated with TreeView) generated from multi-sequence alignment of the intergenic region of *aln* from 62 isolates demonstrates that nearly all of the rough isolates phylogenetically cluster, and are distinct from smooth isolates of *A*. *haemolyticum*. * Represent outliers. Previously designated as smooth, strain S21 was shown in our laboratory to be a rough isolate and was re-designated as R21 and phylogenetically clusters with other rough isolates. Black line separates rough isolates (top) from smooth isolates (bottom). 11 isolates not shown in the tree are rough isolate R31, smooth isolate B5366 and the nine isolates from Denmark and Germany. These isolates also cluster in the tree as rough or smooth (data not shown for 11 isolates to prevent overcrowding of figure).

Remarkably, the upstream region of *aln* from nearly all of the rough biotypes were phylogenetically clustered, and were distinct from the *aln* upstream regions of smooth biotypes (Fig 4A). This upstream region allowed for 90% (66/73) of the strains to be clearly separated as either smooth or rough. The only outlier strains are S19, R29, R48, B0961, 7–4438845, 7–2596628, and P646. Strains B1025 and B4636 have smooth colony morphology and are at the boundary between smooth and rough in the phylogenetic tree. Strain B0961 is also close to the smooth-rough boundary in the tree.

We conducted part of the study in a blind fashion to determine whether we could accurately predict smooth versus rough based solely on sequence analysis of the *aln* upstream region. In the first experiment, all of the United States isolates were sequenced in the *aln* upstream region by two investigators (MPW and AC) and another investigator (DJM) conducted the colony morphologies. Twelve of 13 strains were correctly distinguished as either smooth or rough by the two methods and correlated with each other. The lone outlier was strain B0961, which has a rough colony morphology but the *aln* upstream region clusters with the smooth isolates in the tree, but it is close to the smooth-rough boundary in the tree.

In the second blind experiment, chromosomal DNA from the nine *A*. *haemolyticum* Denmark and German isolates was received in the United States and the *aln* upstream region was sequenced and all nine were found to phylogenetically cluster as smooths. In a blind fashion, two of us (OS and CL) determined colony morphology. Six of nine of these isolates had the smooth colony morphology (the three rough were 7–4438845, 7–2596628 and P646). Thus, in the two blind experiments, we accurately predicted the colony morphology in 18/22 (82%) of the isolates, after we had the sequence data from the *aln* upstream region.

**Fig 5:**
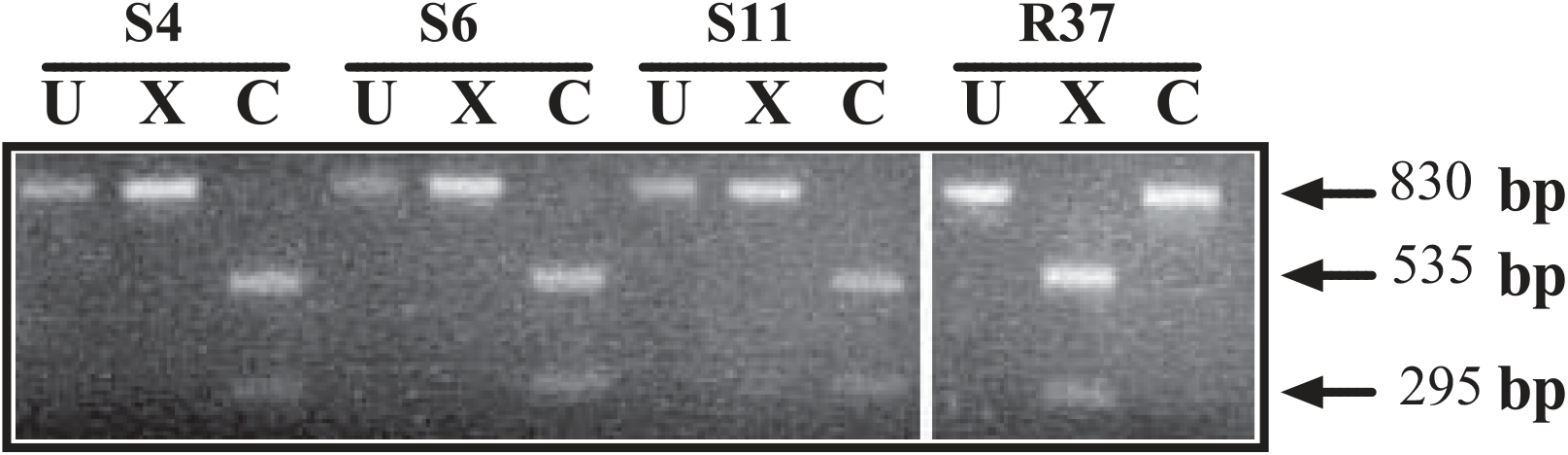
Smooth and rough clinical isolates of *A*. *haemolyticum* can be distinguished by differential restriction enzyme analysis of the upstream region of *aln*. The upstream region of *aln* was PCR-amplified using primers DM1078 and DM1080 to generate an ~830 bp product. Purified PCR products were cleaved with *ClaI* or *XcmI*. Representative of 21 strains tested. *In silico* results of all 73 strains shown in Table 3. U, undigested; C, *ClaI*, X, *XcmI*. Both single digests yield products of about 295 bp and 535 bp. Undigested product is ~830 bp.

Inspection of the sequence data from the *aln* upstream region revealed predicted differential restriction enzyme cleavage patterns (Table 1). The upstream region of *aln* from most rough isolates (32/37) could be cleaved by *Xcm*I but not *Cla*I, whereas the *aln* upstream region from most smooth isolates could only be cleaved by *Cla*I, but not *Xcm*I (27/35) (*P* <0.0001) (Table 3). The *aln* upstream region predicted that five smooth isolates (S15, S24, B5229-01, B5813-02, P5648/10) and one rough isolate (R48) would fail to cut by both enzymes. We verified this *in silico* restriction enzyme differential analysis on twenty-one *aln* upstream region PCR products (representatives shown, Fig 5). This *in silico* predictions held, with the *aln* upstream region from smooth isolates being digested by *Cla*I, rough isolates with *Xcm*I and strains B5813-02, B5229-01, and P5648/10 remained undigested by either restriction enzyme.

**Table 3:** Summary of restriction enzyme patterns of the *aln* upstream region of 73 *A*. *haemoluyticum* clinical isolates based on *in silico* sequence analysis.

Colony Type	Restriction Pattern^1^		
Smooth (S) or Rough (R)	C	X	Neither
R	4	32	1
S	27	4	5

^**1**^ C, *Cla*I; X, *Xcm*I; Neither, neither enzyme digested the upstream region of *aln*. The difference between smooth and rough is statistically significant, *P* < 0.0001. The *aln* upstream region of smooth strains B5813-02, B5229-01, P5648/10l, S15, and S24and rough strain R48 failed to digest with either enzyme. No strain demonstrated an *aln* upstream that digested with both enzymes. The four rough isolates with an *aln* upstream region predicted to digest with *Cla*I were R29, 7–2596628, 7–4438845, and P646. The four smooth isolates with an *aln* upstream region predicted to digest with *Xcm*I were B4636-01, S13, S19 and B1025-98.

**Fig 6:**
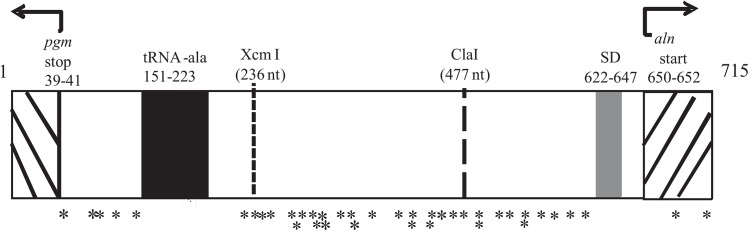
Nucleotide polymorphisms found in the intergenic region of *aln*. Multi-sequence alignment of the ~715 bp intergenic region of *aln* from clinical isolates of *A*. *haemolyticum* shows 40 polymorphisms. Nucleotides 39–41 are the translation stop codon for the upstream gene *pgm*, encoding phosphoglycerate mutase. Nucleotides 151–223 encode the tRNA-ala gene. Nucleotides 633–647 contain the Shine-Dalgarno (SD) sequence, and nucleotides 650–652 are the translation start codon for *aln*. * Denote polymorphisms detailed in Table 4.

Finally, there were 40 nucleotide polymorphisms found within the upstream region of *aln* (Fig 6 and Table 4). 12 of these polymorphisms were only found in strains P646 and 2289/09 and the significance of that is unclear. Two other polymorphisms were found only in one strain. Remarkably, 20 of the 26 remaining polymorphisms highly correlated with the smooth versus rough phenotypes (Table 4). For example, at position 125 all rough isolates had a C whereas 29 smooth isolates had a T and 10 had a C (*p* = 0.0001). Moreover, nucleotides corresponding to the *Xcm*I site (position 236) and *Cla*I site (position 477) featured polymorphisms that were highly correlative with smooth (*Cla*I) versus rough (*Xcm*I), as previously demonstrated above. We conclude that the sequence data from the upstream region of *aln* can be used as a strong predictor to distinguish between rough and smooth biotypes of *A*. *haemolyticum*.

**Table 4:** Nucleotide polymorphisms in *A*. *haemolyticum aln* upstream region. Numbering based on ATCC9345. Nucleotides 39–41 are the translation stop codon for the upstream gene, *pgm*, encoding a phosphoglucomutase. Nucleotides 151–223 encode the tRNA-ala, which is completely conserved in all isolates. Nucleotides 633–647 contain the Shine-Dalgarno sequence, and 650–652 is the translation start codon for *aln*.

Nucleotide #	Polymorphism	Roughs	Smooths	Comment	R vs S *p* value
42 ^a^	G or A	36 A	35 A		1.0
		1 G	1 G		
97 ^a^	G or A	36 G	35 G		1.0
		1 A	1 A		
101	G or A	34 G	36 G	A only in strains R29, 7–2596628 and 7–4438845	1.0
		3 A	0 A		
117	G or A	34 G	36 G	A only in strains R29, 7–2596628 and 7–4438845	1.0
		3 A	0 A		
125	C or T	1 T	28 T		0.0001
		36 C	8 C		
230	G or C	36 G	32 G	All strains with C belong to same subclade; R48 is rough outlier grouping with smooth isolates	1.0
		1 C	4 C		
236	G, T, or C	32 T	4 T	XcmI sitel	G-T 0.0001
		4 G	31 G		
		1 C	1 C		
242 ^a^	C or A	36 C	35 C		0.50
		1 A	1 A		
244 ^a^	G or A	36 A	35 A		0.50
		1 G	1 G		
265	A or G	36 A	32 A	Rough with G is outlier strain R48 and forms subclade with the 4 smooth isolates with G	0.36
		1 G	4 G		
267	T or G	37 T	24 T		0.0002
		0 G	12 G		
281 ^a^	T or A	36 T	35 T		0.50
		1 A	1 A		
291	C or T	32 C	16 C		0.0001
		5 T	20 T		
295	A or G	35 A	19 A		0.0001
		2 G	17 G		
298	G or A	35 G	19 G		0.0001
		2 A	17 A		
299	A or G	36 A	32 A	Rough isolate with G is outlier strain R48	0.36
		1 G	4 G		
314	T or C	36 T	20 T	Rough isolate with C is outlier strain R48	0.0001
		1 C	16 C		
317	G or A	33 G	22 G		0.0001
		4 A	14 A		
318	C or T	36 C	32 C	Rough isolate with T is outlier strain R48	0.36
		1 T	4 T		
328	A or C	36 A	36 A	Only in strain R35	1.0
		1 C	0 C		
360	A, G, or T	3 G	15 G		G-T 1.0
		0 T	12 T		G-A 0.0001
		34 A	9 A		A-T 0.0001
408	C or T	34 C	21 C		0.0001
		3 T	15 T		
409 ^a^	G or A	36 G	35 G		0.50
		1 A	1 A		
438	C or T	32 T	5 T		0.0001
		5 C	31 C		
439	Deletion or T or C	32 deletion	5 deletion	C only in strain 2289/09	0.0001
		5 T	30 T		
		0 C	1 C		
446–456 region	A_6_GC_4_	32	5		0.0001^b^
	A_5_GC_3_	0	15		
	A_5_GC_2_T	1	4		
	A_5_GC_4_	1	3		
	A_5_G_2_C_3_	0	1		
	A_4_G_2_C_4_	3	8		
469 ^a^	T or G	36 T	35 T		0.50
		1 G	1 G		
477	C or T	33 C	9 C	ClaI site	0.0001
		4 T	27 T		
511	T or A	34 T	25 T	Pattern same as nucleotide 539	0.0009
		3 A	11 A		
512	A or G	32 A	20 A		0.0001
		5 G	16 G		
526	T, G, or C	34 T	26 T		T-G 0.007
		3 G	8 G		
		0 C	2 C		
539	T or C	34 T	25 T	Pattern same as nucleotide 511	0.0009
		3 C	11 C		
542 ^a^	G or A	36 G	35 G		0.50
		1 A	1 A		
546 ^a^	G or A	36 G	35 G		0.50
		1 A	1 A		
558	G or C	34 C	25 C		0.0079
		3 G	11 G		
585	G or A	37 G	24 G		0.0008
		0 A	11 A		
587 ^a^	A or G	36 A	35 A		0.50
		1 G	1 G		
611 ^a^	C or T	36 C	35 C		0.50
		1 T	1 T		
682	C or T	36 C	24 C	In *aln* coding region; does not alter amino acid sequence	0.0001
		1 T	12 T		
712 ^a^	C or T	36 C	35 C	In *aln* coding region; does not alter amino acid sequence	0.50
		1 T	1 T		

^a^ These polymorphisms only present in strains P646 and 2289/09.

^b^ Only top two sequences compared for *p* values.

## Discussion

In 1994, Carlson *et al*. classified *A*. *haemolyticum* into two distinct biotypes, each displaying its own colony morphology, β-hemolysis, and disease association. Smooth isolates possess smooth colony edges, are moderate to strong in hemolytic activity, and are associated with wound infections [9]. In contrast, rough isolates possess rough and irregular colony edges with a “fried egg” appearance, have weak to no β-hemolytic activity, and are associated with pharyngitis [9]. Smooth-rough colony types within a species is found in both Gram-positive and Gram-negative bacteria. In Gram negative bacteria, rough colony morphology is often due to a defective lipopolysaccharide (LPS) layer of the cell, usually resulting in a loss of virulence. Gram-positive bacteria lack this LPS cell envelope layer, so the structural basis of the two *A*. *haemolyticum* biotypes remains to be elucidated. While presence of capsules can contribute to colony morphology, we did not observe capsules in smooth or rough biotypes using the India ink method under the growth conditions tested (data not shown). Carlson and co-workers also did not observe any difference in smooth versus rough organisms by electron microscopy [9].

Identification of *A*. *haemolyticum* in clinical samples includes the use of PCR analysis, alpha-mannosidase, beta-hemolysis, Gram-stain, catalase (-), and reverse CAMP tests (+) [2, 4, 9, 16]. Currently, the only method to distinguish between the two biotypes of *A*. *haemolyticum* is the use of the β-glucuronidase test, in which smooth biotypes are β-glucuronidase negative and rough biotypes are β-glucuronidase positive. There are no molecular distinguishing characteristics between these two biotypes. This work presented herein provides that first genotypic method to distinguish between smooth and rough biotypes of *A*. *haemolyticum*. Notably, this organism is often missed in clinical specimens since hemolysis is weak on sheep blood agar and the organism has a diphtheroids-like appearance reminiscent of normal flora bacteria. This would undoubtedly lead to an under-estimation of the number of cases in human specimens (discussed in [10]). For these reasons, it is difficult to obtain clinical isolates.

For this study, we investigated *aln* sequence variation and hemolytic activity between rough and smooth isolates of *A*. *haemolyticum*. PCR amplification of the *aln* ORF from smooth isolates yielded the predicted 2.0 kb product. Surprisingly, we found that amplification of the *aln* ORF from some rough isolates resulted in a 3.2 kb product that contains an IS element with homology to a transposase and integrase from *C*. *diphtheriae*. We found that none of the United States and Denmark isolates contained this IS element; however half of the rough isolates from Finland possessed this IS element. This difference could possibly be due to geographical location and year of acquisition of the isolates, both of which could contribute to the evolution of the organism. Analyzing *A*. *haemolyticum* isolates from other geographical locations from different outbreaks could help to address whether geographic location impinges on IS presence within the *aln* coding region. The location of the IS element could explain why some rough isolates are poorly hemolytic; however, the presence or absence of the IS element in the rough isolates may not be the only factor contributing to the hemolytic activity of the organism, since most rough isolates tend to be weakly hemolytic, even when the IS element is absent from the *aln* coding region. Also, the *aln* gene would need to be disrupted in smooth and rough isolates to determine whether all of the hemolysis is solely due to *aln* or to other unidentified hemolysins. Since half of the rough isolates lack the IS element within *aln* yet tend to have low hemolytic activity, variable regions in the *aln* upstream region may also modulate hemolytic activity. Other areas of the genome unique to rough isolates may also be responsible for low hemolytic activity of rough isolates. All rough isolates containing the IS element within *aln* had the IS element in the same location, suggesting the insertion is not a random event. We propose that there is specificity to the IS insertion, but the mechanism remains undefined. Whether the IS transposase and integrase are functional still remains to be elucidated. Nonetheless, it appears that there are now at least two major subtypes of rough isolates- those that carry the IS element within the *aln* coding region and those that do not. As further genetic loci are molecularly characterized, it may be possible to discover additional subtypes of rough and smooth isolates.

We also found ~40 nucleotide polymorphisms in the intergenic region of *aln*. Interestingly, this region allowed for distinct phylogenetic clustering to occur between smooth and rough isolates of *A*. *haemolyticum*. Some isolates were outliers within the tree; however, when these isolates were streaked on TH horse blood agar plates, we confirmed that all isolates had the predicted colony morphology. The lone exception is AhR21, which was previously designated by Carlson and colleagues as a smooth isolate (AhS21) [9], but in our laboratory shows a rough morphology that led us to rename the strain. It is unknown why several of the isolates are outliers in the tree, and it suggests that additional genetic determinants may allow for the prediction of smooth versus rough morphologies in a subset of *A*. *haemolyticum*. Nonetheless, ~90% of the strains were clearly separated as smooth versus rough using the sequence from the upstream region of *aln*.

Given the heterogeneity of the upstream region of *aln* in this small number of clinical isolates from limited geographic locations, we postulate that this organism will have more genetic heterogeneity in other loci awaiting discovery.

This sequence variation also allowed for differential restriction enzyme cleavage patterns to occur. For example, the intergenic region from smooth isolates can be cleaved by *Cla*I but not *Xcm*I, whereas the intergenic region from rough isolates can be cleaved by *Xcm*I but not *Cla*I. This differential restriction enzyme cleavage was observed for nearly all *A*. *haemolyticum* isolates except for B5813-02, B5229-01, and P5648/10. All Denmark isolates have an *aln* upstream region that clusters with smooth isolates, but the live strains were not available in our laboratory to confirm the colony morphology.

The very strong correlation between the *aln* upstream sequence and whether the isolate is smooth or rough leads to the intriguing hypothesis that the *aln* upstream region more directly contributes to the smooth/rough phenotype. Future work will focus on determining whether there is a direct causal relationship between the *aln* upstream sequence variation and associated corresponding colony morphology and what role the IS element plays in hemolysis. This study provides the first molecular tool to distinguish between smooth and rough biotypes of *A*. *haemolyticum*, an important emerging bacterial pathogen of humans.
